# Dynamic Regulation of Circulating microRNAs During Acute Exercise and Long-Term Exercise Training in Basketball Athletes

**DOI:** 10.3389/fphys.2018.00282

**Published:** 2018-03-26

**Authors:** Yongqin Li, Mengchao Yao, Qiulian Zhou, Yan Cheng, Lin Che, Jiahong Xu, Junjie Xiao, Zhongming Shen, Yihua Bei

**Affiliations:** ^1^Cardiac Regeneration and Ageing Lab, School of Life Science, Shanghai University, Shanghai, China; ^2^Department of Psychiatry, Tongji Hospital of Tongji University, Shanghai, China; ^3^Department of Cardiology, Tongji Hospital, Tongji University School of Medicine, Shanghai, China

**Keywords:** acute exercise, long-term exercise, basketball athletes, circulating miRNAs, cardiovascular adaptation

## Abstract

Emerging evidence indicates the beneficial effects of physical exercise on human health, which depends on the intensity, training time, exercise type, environmental factors, and the personal health status. Conventional biomarkers provide limited insight into the exercise-induced adaptive processes. Circulating microRNAs (miRNAs, miRs) are dynamically regulated in response to acute exhaustive exercise and sustained rowing, running and cycling exercises. However, circulating miRNAs in response to long-term basketball exercise remains unknown. Here, we enrolled 10 basketball athletes who will attend a basketball season for 3 months. Specifically, circulating miRNAs which were involved in angiogenesis, inflammation and enriched in muscle and/or cardiac tissues were analyzed at baseline, immediately following acute exhaustive exercise and after 3-month basketball matches in competitive male basketball athletes. Circulating miR-208b was decreased and miR-221 was increased after 3-month basketball exercise, while circulating miR-221, miR-21, miR-146a, and miR-210 were reduced at post-acute exercise. The change of miR-146a (baseline vs. post-acute exercise) showed linear correlations with baseline levels of cardiac marker CKMB and the changes of inflammation marker Hs-CRP (baseline vs. post-acute exercise). Besides, linear correlation was observed between miR-208b changes (baseline vs. after long-term exercise) and AT VO_2_ (baseline). The changes of miR-221 (baseline vs. after long-term exercise) were significantly correlated with AT VO_2_, peak work load and CK (after 3-month basketball matches). Although further studies are needed, present findings set the stage for defining circulating miRNAs as biomarkers and suggesting their physiological roles in long-term exercise training induced cardiovascular adaptation.

## Introduction

Systematic physical exercise is an effective clinical option to improve quality of life in many types of patients, and the beneficial effects of exercise on cardiac function and exercise capacity have been well documented. It is well established that regular physical exercise promotes the improvement of life quality and lengthens lifespan (Moore et al., 2012; Arem et al., 2015). Furthermore, exercise has been reported to effectively reduce cardiovascular morbidity and mortality in patients with heart diseases (Gayda et al., 2016; Ribeiro et al., 2017). The augment of exercise capacity needs cumulative adaptation of the circulatory, pulmonary, and musculoskeletal systems, and exercise capacity has become an important indicator of cardiovascular health (Bassett and Howley, 2000; Wilson et al., 2016).

Exercise training could impact multiple signaling pathways, which influence energy metabolism, inflammation, regeneration and remodeling of myocardial and skeletal muscle (Egan and Zierath, 2013; Tao et al., 2015). However, whether exercise causes benefits depends on the intensity, training time, exercise type, environmental factors and the personal health status. Increases of circulating biomarkers of destruction parameters [Creatine kinase (CK), Lactic dehydrogenase (LDH)], cardiac markers [Creatine kinase-MB isoenzyme (CKMB), Troponin T and NH2-terminal prohormone of brain natriuretic peptide (NT-proBNP)], inflammatory marker [high-sensitivity C-reactive protein (Hs-CRP)] have been well documented (Clyne and Olshaker, 1999; Rifai et al., 1999; Bhalla et al., 2004; Hekimsoy and Oktem, 2005). However, these biomarkers provide limited insight into the exercise-induced adaptive processes. Thus, new biomarkers capable of monitoring cardiac changes and evaluating exercise physiology are critically needed.

MicroRNAs (miRNAs, miRs) are endogenous small noncoding RNAs and range from 18 to 25 nucleotides in length. miRNAs regulate gene expression at post-transcriptional levels through inhibition of translation or mRNA degradation in sequence-dependent manner (Guo et al., 2010). miRNAs have been widely detected in various human tissues, playing pivotal roles in a wide range of physiological and pathological processes. Besides tissues, miRNAs can also be detected in body fluids such as serum, plasma, urine, and saliva (Gautam et al., 2016). Circulating miRNAs are stable, easily detectable, and sensitive response to health changes, which endow the plasma and /or serum miRNAs with the potential use as biomarkers.

To date, increasing evidence has reported circulating miRNAs as potential therapeutic biomarkers for cardiovascular diseases, and numerous circulating miRNAs are found to be associated with exercise both in healthy human and patients. Numerous cardiac and/or skeletal muscle enriched miRNAs related with muscle damage, inflammation, cardiac adaptation, and angiogenesis have been shown altered after acute and/or sustained exercise (Baggish et al., 2011; Xu et al., 2016), indicating that circulating miRNAs can strongly monitor exercise influence on pathological and physiological processes of cardiovascular and muscles. Profiles of circulating miRNAs are varying under different exercise type, intensity and duration time. Dynamic regulation of circulating miRNAs response to acute and sustained exercise has been deciphered both in healthy participants and athletes (Baggish et al., 2011; Nielsen et al., 2014; Denham and Prestes, 2016). However, the intensity of long-term exercise training performed in previous studies is moderate, and regarding the energy metabolism system, nearly all the exercise type in previous studies is aerobic exercise (running, cycling, ski, and rowing etc.) (Baggish et al., 2011; Aoi et al., 2013; Tonevitsky et al., 2013; Nielsen et al., 2014; Uhlemann et al., 2014; Schmitz et al., 2017) or anaerobic exercise (sprint interval cycling; Cui et al., 2015). It is still unknown whether and how the mixed anaerobic-aerobic exercise with high intensity (basketball, football, volleyball, and handball etc.) influences circulating miRNAs and cardiac adaptation.

Here, we inspect how acute and long-term exercise training affects specific circulating miRNAs with well-known roles in adaptive process in basketball athletes. Specifically, we determined the change of circulating miRNA levels, general echocardiographic indexes, biochemical parameters and cardiorespiratory fitness at baseline and after a 3-month basketball season. Besides, the circulating miRNA levels and biochemical parameters are also assessed during acute exercise testing. We found that cardiorespiratory fitness and inflammation markers are significantly increased without the change of general echocardiographic indexes after 3-month basketball matches. Circulating miR-208b was decreased and miR-221 was increased after long-term exercise, while circulating miR-221, miR-21, miR-146a, and miR-210 were decreased response to acute exercise. Moreover, we correlated the circulating miRNA changes with biochemical parameters and cardiorespiratory fitness, showing the potential of circulating miRNA as biomarkers of initial stress induced by acute exercise and adaptation response to long-term basketball exercise.

## Materials and methods

### Participants

This study was carried out in accordance with the recommendations of the ethics committee of Shanghai University. All subjects gave written informed consent in accordance with the Declaration of Helsinki. Ten male basketball athletes planning to participate in 3-month amateur basketball matches were enrolled in this study. The average playing time of every athlete is 260 min during the 3 months. And all except one also perform the regular exercise training such as fitness and playing basketballs during the intermission period after matches, and the average regular training time of every athlete is 7,341 min in 3 months. Clinical characteristics and echocardiographic indexes were recorded at the initiation time of enrollment and the echocardiographic indexes were also measured after 3-month basketball matches.

### Serum sampling

At baseline, immediately after the cardiopulmonary exercise and 3-month after basketball season, venous blood was collected in silicone-coated serum tubes with increased silica act clot activator, followed by processing within 1 h after collection. At 4°C, blood samples were centrifuged at 3,000 rpm for 15 min. The supernatant (serum) was collected into RNase/DNase-free tubes and immediately aliquoted and frozen at −80°C.

### Cardiopulmonary exercise test

A cardiopulmonary exercise testing was performed before and after the 3-month basketball season. All participants were refrained from any physical exercise for 1 day before testing and were conducted an overnight dietary fast. On the morning of testing, participants were encouraged to drink water (400–800 ml) without caloric or electrolyte content. Ten basketball athletes underwent a cardiopulmonary exercise test on a MasterScreen-CPX system (Jaeger, Germany) at Shanghai Tongji Hospital (Baggish et al., 2011). Briefly, following a 3 min period of rest and a 3-min unloaded exercise, the workload was increased at a pre-set rate (starting from 0 J/s for 1 min and increased by 2 J/s every 6 s). The subjects continued pedaling at 60~70 rpm throughout the test, until they reached their peak oxygen consumption.

### Biochemical measurements

Creatine kinase (CK), CKMB, Troponin T, NT-proBNP, and Hs-CRP were measured by enzyme linked immunosorbent assays (ELISA) kits from Wuhan Xinqidi Biological Technology. Lactic dehydrogenase (LDH) was determined by ELISA kits (KeyGEN BioTECH).

### RNA isolation

The total RNA extraction was performed using a mirVanaPARIS isolation kit (Ambion, Austin, Texas) according to the manufacturer's instructions. To avoid variability of results, repeated freeze-thaw cycles of serum samples were minimized and all samples were extracted and analyzed in a single batch. Briefly, 400 μl of serum was used to extract the total RNA. After equal volume of denaturing solution was added, 50 pmol/L Caenorhabditis elegans miR-39 (cel-miR-39) was added to normalize the miRNA serum levels.

### Quantification of circulating miRNA levels

To quantify circulating miRNA levels, Bulge-Loop™ miRNA qPCR Primer Sets (RiboBio) and quantitative reverse transcription polymerase chain reactions (qRT-PCRs) with Takara SYBR Premix Ex Taq^TM^ (TliRnaseH Plus) were used to detect selected miRNA expressions. All qRT-PCR reactions were performed in triplicate using an Applied Biosystems 7900HT Fast Real Time PCR device. Fold-change of RNA species was calculated using the formula 2^(−ΔΔ Ct)^ and Cel-miR-39 were used as spike-in control.

### Statistical analysis

Subject characteristics, biochemical measurements and general echocardiographic indexes are presented as means ± standard error of the mean (SEM). Paired-samples *t*-test was performed to analyze the changes of miRNAs, general echocardiographic indexes, biochemical indexes and cardiorespiratory fitness before and after 3-month exercise. Correlation analyses were analyzed using the Spearman's or Pearson's method as appropriate for data distribution. Statistical significance is defined as *P*-values < 0.05.

## Results

### Baseline and post-training subject characteristics

**Figure 1:**
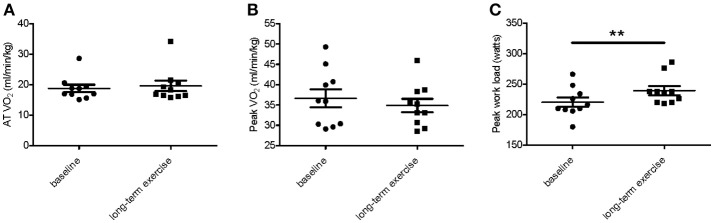
Peak work load increased in response to long-term exercise training. At baseline and after 3-month matches, the parameters of cardiopulmonary exercise testing AT VO_2_ **(A)**, peak VO_2_ **(B)**, and peak work load **(C)** were measured in 10 male athletes (*n* = 10). Values are presented as statistical means, and error bars show SEM; ^**^P < 0.01.

Ten male basketball athletes of one team were selected as our subjects, before they participated in an amateur basketball season for 3 months. And the clinical characteristics of athletes at the baseline were shown in Table 1. Their mean age is 25.90 ± 4.95 years, mean BMI is 25.88 ± 2.46, and the average heart rate is 72.50 ± 7.98 beats/min. Table 2 lists the detailed general echocardiographic indexes of baseline and after 3-month exercise, and there was no significant difference in all echocardiographic parameters. We analyzed the biochemical measurements of 10 athletes at baseline, post-acute exercise and long-term exercise training (Table 3). The results showed that there were no significant changes of destruction parameters (CK, LDH), cardiac markers (CKMB, Troponin T, and NT-ProBNP) after post-acute exercise and long-term exercise training, while inflammatory marker Hs-CRP was significantly up-regulated after long-term exercise training. Cardiopulmonary exercise tests revealed that though the AT VO_2_ and peak VO_2_ were not changed (Figures 1A,B), the peak work load increased significantly after long-term exercise training (Figure 1C).

**Table 1:** Clinical characteristic of participants.

**Clinical parameters**	**Mean ± SEM**
Age (years)	25.90 ± 4.95
Height (cm)	185.30 ± 4.74
Body mass (kg)	89.00 ± 10.25
BMI (kg/m^2^)	25.88 ± 2.46
Heart rate (beats/min)	72.50 ± 7.98
Systolic blood pressure (mmHg)	108.50 ± 8.10
Diastolic blood pressure (mmHg)	72.80 ± 10.01

**Table 2:** General echocardiographic indexes.

**Clinical parameters**	**Mean ± SEM**	
	**Baseline**	**After long-term exercise training**
Aortic root dimension (mm)	30.90 ± 3.45	31.40 ± 3.24
Left ventricular end diastolic diameter (mm)	55.20 ± 1.87	54.80 ± 2.39
Left ventricular end systolic diameter (mm)	35.80 ± 2.10	35.60 ± 2.46
End-diastolic volume	148.30 ± 11.60	146.00 ± 15.78
End-systolic volume	54.90 ± 8.88	52.80 ± 11.92
Left atrial dimension (mm)	36.40 ± 3.06	37.20 ± 3.29
Interventricular septal thickness (mm)	9.40 ± 1.26	9.80 ± 0.63
Left ventricular posterior wall thickness (mm)	9.40 ± 1.26	9.70 ± 0.48
Ejection fraction (EF%)	63.30 ± 3.74	64.30 ± 4.69
Fractional shortening (FS%)	34.60 ± 2.55	35.90 ± 3.25

**Table 3:** Biochemical measurements.

	**Baseline**	**Post-acute exercise**	**After long-term exercise training**
**DESTRUCTION PARAMETERS**			
CK (ng/ml)	8.59 ± 0.92	8.36 ± 0.89	7.35 ± 0.94
LDH (ng/ml)	14.06 ± 1.69	15.10 ± 1.63	14.38 ± 1.59
**CARDIAC MARKERS**			
CKMB (ng/ml)	59.99 ± 5.79	65.02 ± 5.82	60.80 ± 5.83
Troponin T (pg/ml)	22.57 ± 2.69	22.42 ± 2.94	26.51 ± 3.40
NT-ProBNP (pg/ml)	103.06 ± 37.36	103.75 ± 41.33	91.04 ± 36.55
**INFLAMMATORY MARKERS**			
Hs-CRP (ng/ml)	3.14 ± 0.28	3.20 ± 0.59	4.06 ± 0.43^*^

Values are means ± SEM.

* *, significant difference between baseline and after long-term exercise training*.

### Circulating mir-208b was decreased and mir-221 was increased after long-term exercise training, while circulating mir-221, mir-21, mir-146a, and mir-210 were decreased post-acute exercise

**Figure 2:**
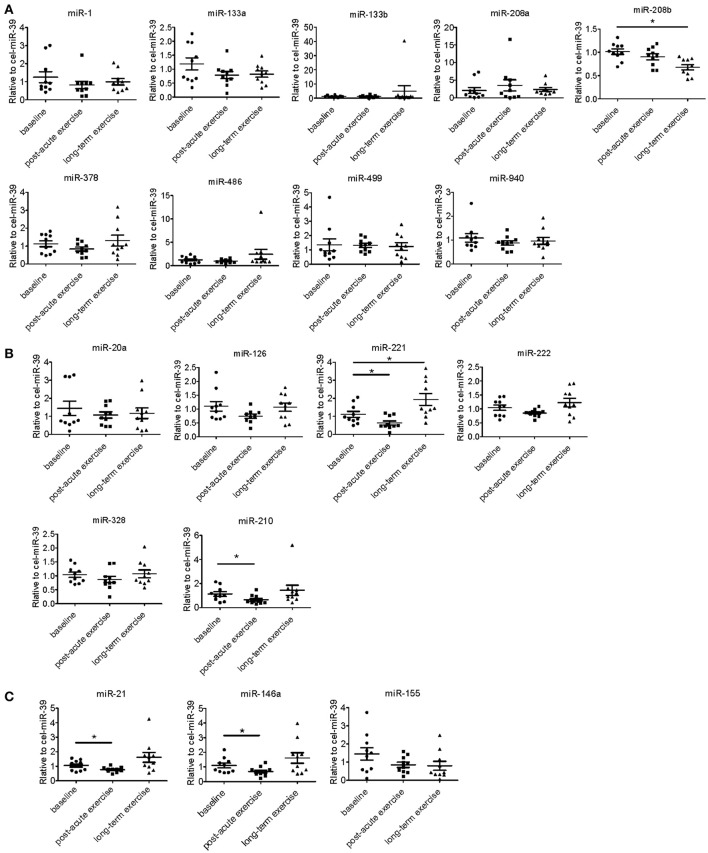
Distinct regulatory profiles of circulating miRNAs after acute exercise and long-term exercise training. **(A)** Serum levels of cardiac or muscle-specific/enriched miRNAs at baseline, post-acute exhaustive exercise and after 3-month basketball match. **(B)** Serum levels of angiogenesis-related miRNAs at baseline, post-acute exhaustive exercise and after 3-month basketball match. **(C)** Serum levels of inflammation-related miRNAs at baseline, post-acute exhaustive exercise and after 3-month basketball match. ^*^P < 0.05; *n* = 10.

We determined the expression of angiogenesis-related miRNAs (miR-20a, miR-126, miR-210, miR-221, miR-222, miR-328; Dews et al., 2006; Poliseno et al., 2006; Kuehbacher et al., 2007; Fasanaro et al., 2008; Soeki et al., 2016), inflammation-related miRNAs (miR-21, miR-146a, miR-155; Taganov et al., 2006; Urbich et al., 2008; Wang et al., 2017) and cardiac or muscle-specific/enriched miRNAs (miR-1, miR-133a, miR-133b, miR-208a, miR-208b, miR-378, miR-486, miR-499, miR-940; Chen et al., 2006; Soci et al., 2016; Xu et al., 2016). The results showed that long-term exercise training significantly decreased serum miR-208b and increased miR-221, while acute exercise decreased circulating miR-221, miR-21, miR-146a, and miR-210 (Figure 2).

### Correlation analysis between miRNA changes following acute exercise and cardiac function, exercise capacity, muscle damage, and inflammation

**Figure 3:**
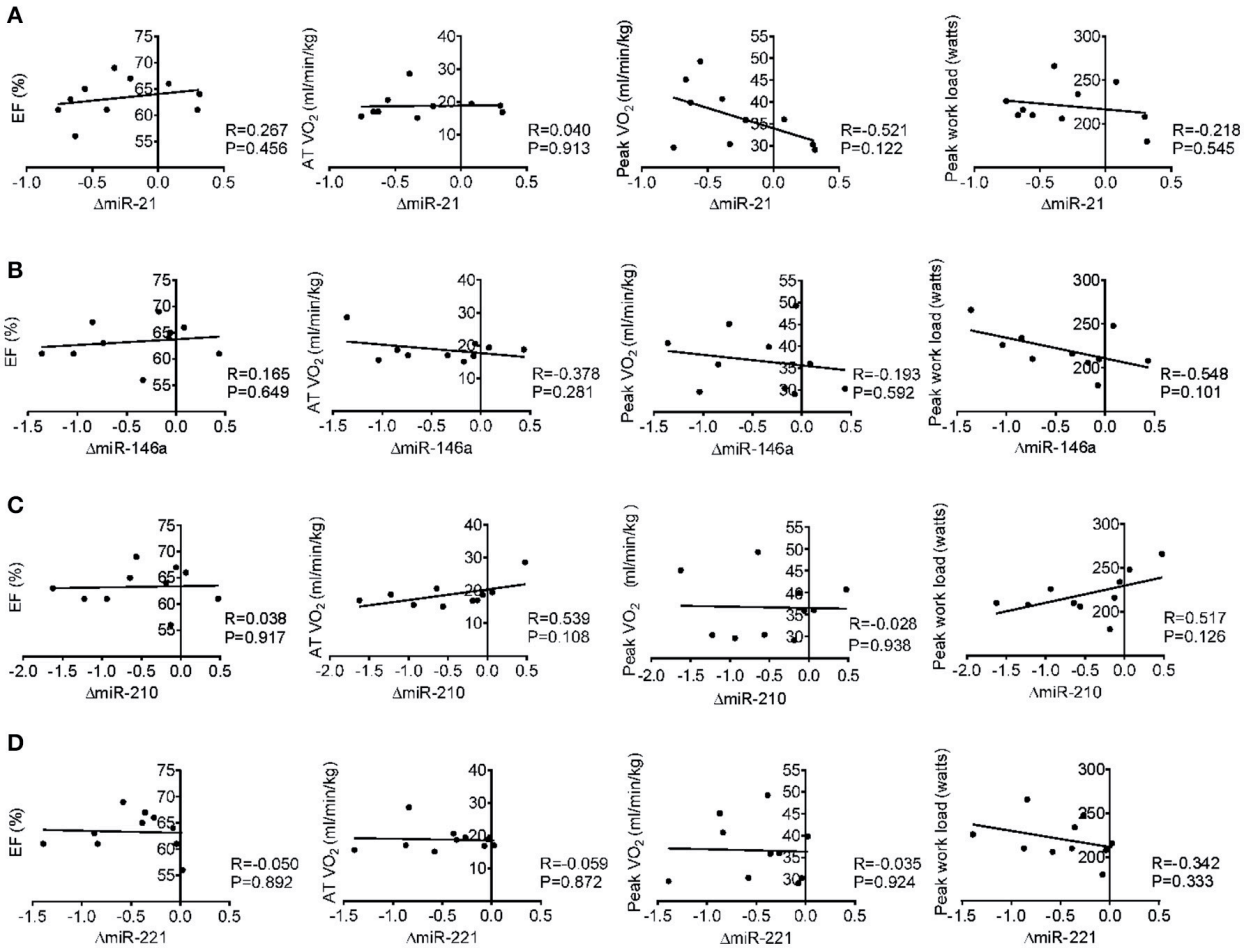
Correlation analysis between the changes of miR-21 **(A)**, miR-146a **(B)**, miR-210 **(C)** and miR-221 **(D)** following acute exercise and cardiac function (EF%), exercise capacity (AT VO_2_, peak VO_2_, and peak work load) at baseline.

Here we correlated the decrease of miR-21, miR-146a, miR-210, and miR-221 after acute exercise with the cardiac function and exercise capacities at baseline, however, no robust correlations were found (Figure 3). Then we analyzed the correlations of miR-21, miR-146a, miR-210, and miR-221 changes after acute exercise with biochemical indexes. While no miRNA showed any significant correlation with CK, LDH, Troponin T and NT-ProBNP, the changes of miR-146a after acute exercise showed significant correlations with baseline levels of cardiac marker CKMB (*R* = −0.703, *P* = 0.023) and the changes of inflammation marker Hs-CRP after acute exercise (*R* = −0.634, *P* = 0.049) (Table 4).

**Table 4:** Correlation analysis between miRNA changes following acute exercise and biochemical indexes.

**ΔmiRNA**		**CK**		**LDH**		**CKMB**		**Troponin T**		**NT-proBNP**		**Hs-CRP**	
		**R**	**P**	**R**	**P**	**R**	**P**	**R**	**P**	**R**	**P**	**R**	**P**
ΔmiR-21	Baseline	−0.32	NS	−0.10	NS	−0.57	NS	0.17	NS	0.25	NS	0.16	NS
	Acute	0.08	NS	0.11	NS	−0.06	NS	−0.04	NS	0.34	NS	−0.22	NS
	Δ	0.35	NS	0.56	NS	0.62	NS	−0.12	NS	0.59	NS	−0.23	NS
ΔmiR-146a	Baseline	−0.43	NS	−0.25	NS	−0.70	0.02	0.41	NS	−0.03	NS	0.55	NS
	Acute	−0.49	NS	−0.11	NS	−0.40	NS	0.27	NS	0.05	NS	−0.55	NS
	Δ	−0.04	NS	0.41	NS	0.37	NS	−0.07	NS	0.36	NS	−0.63	0.049
ΔmiR-210	Baseline	0.30	NS	−0.16	NS	0.17	NS	0.11	NS	0.04	NS	−0.14	NS
	Acute	−0.04	NS	−0.10	NS	−0.06	NS	−0.51	NS	0.03	NS	0.08	NS
	Δ	−0.30	NS	0.20	NS	−0.29	NS	−0.38	NS	−0.02	NS	0.11	NS
ΔmiR-221	Baseline	−0.37	NS	0.02	NS	−0.60	NS	0.24	NS	0.02	NS	0.23	NS
	Acute	−0.60	NS	0.15	NS	−0.58	NS	0.13	NS	0.06	NS	−0.41	NS
	Δ	−0.19	NS	0.34	NS	0.02	NS	−0.06	NS	0.25	NS	−0.40	NS

Δ, *changes between baseline and post-acute exercise; NS, not significant; Acute, the value of indicated indexes after acute exercise*.

### Correlation analysis between miRNA changes after long-term exercise training and cardiac function, exercise capacity, muscle damage, and inflammation

**Figure 4:**
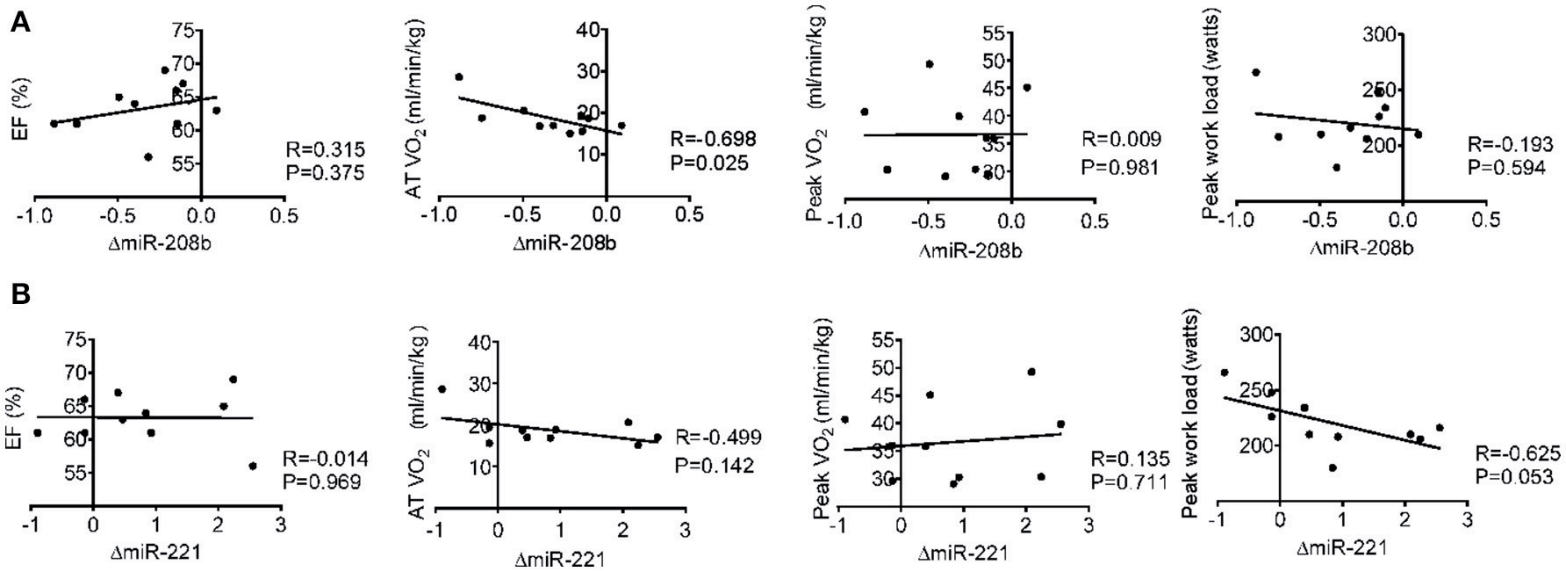
Correlation analysis between the changes of miR-208b **(A)** and miR-221 **(B)** following long-term exercise training and cardiac function (EF%), exercise capacity (AT VO_2_, peak VO_2_, and peak work load) at baseline.

**Figure 5:**
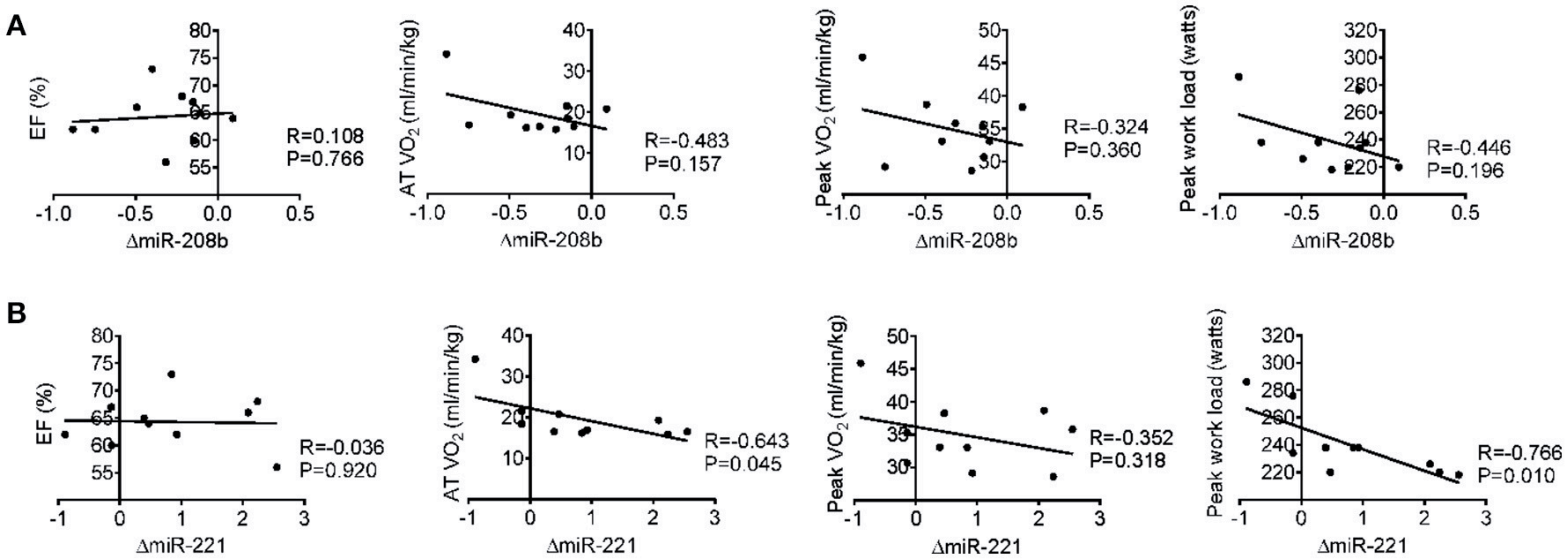
Correlation analysis between the changes of miR-208b **(A)** and miR-221 **(B)** following long-term exercise training and cardiac function (EF%), exercise capacity (AT VO_2_, peak VO_2_, and peak work load) after long-term exercise training.

**Figure 6:**
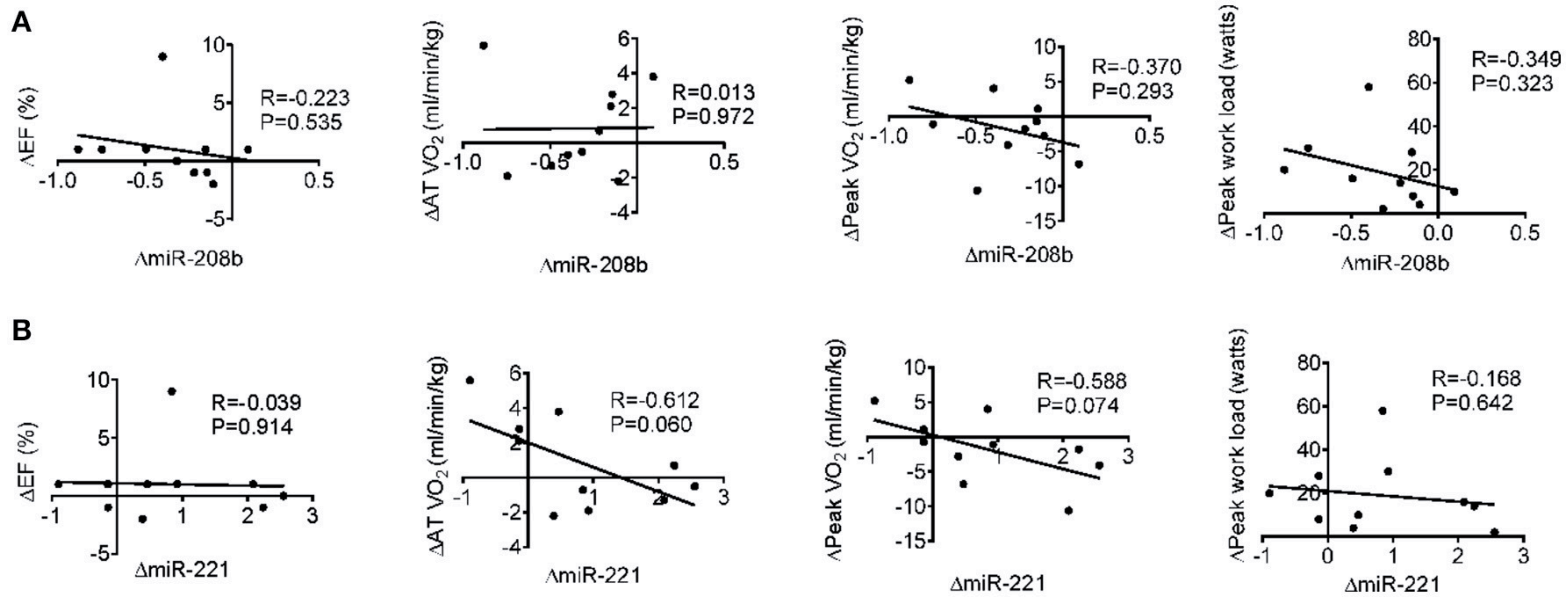
Correlation analysis between the changes of miR-208b **(A)** and miR-221 **(B)** following long-term exercise training and the changes of cardiac function (EF%), exercise capacity (AT VO_2_, peak VO_2_, and peak work load) after long-term exercise training.

Long-term exercise training elevated the expression of miR-221 and decreased the expression of miR-208b (Figure 2). We also correlated the changes of miR-221 and miR-208b induced by long-term exercise training with markers of cardiac function, exercise capacity and indicators of muscle damage and inflammation. The results showed that miR-208b changes induced by long-term exercise training were correlated with the AT VO_2_ at baseline (Figure 4), while miR-221 changes induced by long-term exercise training were significantly associated with the AT VO2 (*R* = −0.643, *P* = 0.045), peak work load (*R* = −0.766, *P* = 0.010) (Figure 5) and CK (*R* = −0.764, *P* = 0.010) (Table 5) after 3-month basketball matches. The miR-208b and miR-221 changes did not show a strong correlation with the other parameters such as EF (%), peak VO_2_, LDH, CKMB, Troponin T, NT-ProBNP, Hs-CRP or their changes (Figures 4–6 and Table 5).

**Table 5:** Correlation analysis between miRNA changes following long-term exercise training and biochemical indexes.

**ΔmiRNA**		**CK**		**LDH**		**CKMB**		**Troponin T**		**NT-proBNP**		**Hs-CRP**	
		**R**	**P**	**R**	**P**	**R**	**P**	**R**	**P**	**R**	**P**	**R**	**P**
ΔmiR-208	Baseline	0.11	NS	0.20	NS	0.44	NS	−0.14	NS	0.09	NS	−0.15	NS
	Long	0.18	NS	−0.12	NS	0.36	NS	0.08	NS	0.02	NS	−0.21	NS
	Δ	0.08	NS	−0.54	NS	−0.14	NS	0.19	NS	−0.43	NS	−0.13	NS
ΔmiR-221	Baseline	−0.30	NS	−0.26	NS	−0.48	NS	0.46	NS	−0.11	NS	0.44	NS
	Long	−0.76	0.01	−0.16	NS	−0.32	NS	−0.12	NS	−0.10	NS	−0.13	NS
	Δ	−0.54	NS	0.19	NS	0.28	NS	−0.50	NS	0.11	NS	−0.49	NS

Δ, *changes between baseline and after long-term exercise; NS, not significant; Long, the value of indicated indexes after long-term exercise*.

## Discussion

The intent of this study is to determine how circulating miRNAs are regulated by an acute exhaustive exercise and long-term exercise training in basketball athletes. Although these athletes were trained regularly before enrolled in our study, their exercise capacities were still enhanced after 3-month exercise training. We reported for the first time, to our best knowledge, that 3 month period of basketball exercise changed miR-208b and miR-221 expression in male athletes. Given that miRNA changes were correlated with biochemical indexes and cardiorespiratory fitness, the present study also provides potential biomarkers for evaluating exercise effects.

To date, the circulating miRNA changes during exercise adaptation in basketball athletes have not been previously published. Competitive basketball is characterized by intermittent exercise with multiple and repeated episodes of high intensity and short duration exertion. The energy demands of basketball were contributed from both aerobic and anaerobic energy systems. These characteristics of basketball make the signature of circulating miRNAs be different from other aerobic and anaerobic exercise types. Our study, for the first time, showed that miR-146a, miR-21, miR-221, and miR-210 were decreased in response to acute exercise in basketball athletes. In addition, our study demonstrated that miR-208b was decreased while miR-221 was increased after the 3-month basketball match in 10 basketball athletes. And other miRNAs (miR-1, miR-21, miR-222, miR-486, miR-146a, and miR-126, etc.) have no changes after 3-month basketball match, which have been shown dynamically altered after different programs of long-term aerobic exercise training (Baggish et al., 2011; Aoi et al., 2013; Nielsen et al., 2014; Denham and Prestes, 2016; Schmitz et al., 2017). Considering most of the previous studies focused on aerobic or anaerobic exercise, our results from mixed aerobic-anaerobic exercise provided novel insights into exercise physiology.

miR-21, miR-146a, and miR-210 regulate multiple functions relevant to cardiovascular diseases and exercise. It can be speculated that acute exercise might cause local short-term hypoxic conditions in skeletal muscle and cardiovascular systems. miR-21 plays anti-apoptosis roles in endothelial cells (Weber et al., 2010) and cardiomyocytes under ischemia (Dong et al., 2009). miR-210 has been found induced by hypoxia in a variety of ischemic conditions and tissues (Bostjancic et al., 2009; Biswas et al., 2010; Hu et al., 2010). By targeting several transcripts, miR-210 regulates multiple aspects of cellular response to hypoxia such as inhibiting apoptosis (Fasanaro et al., 2008; Zaccagnini et al., 2014), promoting the shift from mitochondria respiration to glycolysis (Chan et al., 2009) and inducing angiogenesis (Fasanaro et al., 2008; Alaiti et al., 2012). Thus, the decrease of miR-21 and miR-210 in present study may reflect the initial cellular response to hypoxia after acute exercise, such as increased cell apoptosis and enhanced mitochondrial metabolism. In addition, inflammatory response is also another process accompanied with exercise, especially acute exercise with high intensity. Both miR-21 (Sheedy, 2015), miR-221 (Corsten et al., 2015) and miR-146a (Yang et al., 2012; Cheng et al., 2013) have been identified as vital brake in inflammatory response. It can be speculated that the decrease of miR-21, miR-221 and miR-146a in present study may reflect the initial pro-inflammatory process that occur after acute exercise. Among them, time course of plasma miR-21 level has showed that miR-21 was downregulated immediately after acute exercise while increased during recovery in healthy young men (Cui et al., 2017). Thus, in the future, time course study of circulating miR-21, miR-146a and miR-221 levels will demonstrate the potential roles of circulating miRNAs in real-timely reflecting the inflammatory responses after acute exercise in basketball athletes or other similar types of athletes.

Long-term exercise training causes various adaptations including angiogenesis, mitochondrial metabolism, skeletal muscle regeneration and myocardial remodeling, which will directly interfere with the exercise capacity and health outcomes. miR-208b is expressed in skeletal muscle and embryonic hearts, and its ectopic expression caused multiple cardiovascular diseases (Huang and Li, 2015). Aerobic exercise reduces miR-208b expression and modulates the expression of miR-208b downstream genes such as cardiac myosin heavy chain (MHC) isoforms, PPARβ, and histone deacetylase (HDAC) class I and II, which are associated with cardiac metabolic and contractile adaptation (Soci et al., 2016). The decrease of miR-208b may contribute to the maintenance of physiological cardiac hypertrophy and improvement of cardiac function after exercise training (Soci et al., 2016). miR-221 shows vital roles in vasculature network by inhibiting the proliferation of endothelial cells (Poliseno et al., 2006) and supporting the vascular smooth muscle cell dedifferentiation, proliferation and migration (Liu et al., 2009; Zampetaki and Mayr, 2012). miR-221 has also been shown promoting skeletal muscle regeneration through modulating muscle cell differentiation and maturation (Cardinali et al., 2009; Togliatto et al., 2013). Angiogenesis and muscle regeneration are all key aspects of exercise adaptation. Therefore, miR-221 elevation after long-term exercise training might represent the enhanced muscle regeneration and controlled angiogenesis. Considering miR-221 was the only circulating miRNA in response to both acute and long-term exercise training, findings from this study set the stage for identifying miR-221 as a promising biomarker capable of evaluating the magnitude of stress and adaptation induced by exercise.

In our study, after 3-month exercise training, echocardiography showed that EF% value and other cardiac indexes were not changed, whereas their exercise capacities are enhanced. These results indicate that echocardiography and biochemical measurements are unable to comprehensively evaluate the benefits of long-term exercise training. The biochemical measurements showed that the contributions of both acute and 3-month basketball match to tissue damage does not appear to be significant in the present study. To further determine the uniqueness of circulating miRNA changes, we next compared circulating miRNA profiles to those of conventional biomarkers such as EF%, AT VO_2_, peak VO_2_ and biochemical indexes. With the improvement of exercise capacities, we showed the changes of miR-208b after 3-month exercise training moderately correlated with AT VO_2_ at baseline and the changes of miR-221 correlated with AT VO_2_, peak work load and serum CK levels after 3-month exercise training. Thus, these results indicated the possibility of circulating miR-221 and miR-208b as potential biomarkers for assessing or revealing the muscle damage and adaptations of exercise capacity induced by long-term basketball exercise.

As an initial study, several limitations of this study were still existent. Firstly, considering the variations of exercise intensity and duration time of every subject during the basketball game, the number of enrolled athletes should be expanded. Secondly, in view of the difference of exercise abilities between male and female, it would be interesting to see the miRNA changes in response to long-term exercise training using female athletes as subjects. Thirdly, the current study was restricted to basketball athletes. Future study is required to determine whether the changes of circulating miRNAs are applicable to football or other similar exercise types. Fourthly, our exercise training program involved team-based basketball match. Thus, the volume or intensity of the exercise training could not be controlled and calculated. However, quantitative assessment was taken to document playing time and fitness time of every athlete. Finally, quantitative analysis was restricted to a subset of relevant miRNAs, and high-throughput screening is needed to obtain a more complete profile of circulating miRNA regulation during long-term basketball exercise training.

In conclusion, serum circulating miRNA levels are dynamically regulated in response to acute cycling exercise and long-term basketball exercise training in basketball athletes. Future studies are highly needed to define these miRNAs as potential and useful biomarkers of long-term exercise training, and disclose their direct biological roles in exercise mode-specific training adaptations.

## Author contributions

YL: undertook the qRT-PCR experiments and contributed to the drawing up of figures and writing of the paper; MY: contributed to the drawing up of figures and ELISA experiments; QZ: undertook the qRT-PCR experiments and cardiopulmonary exercise test; YC, LC, and JiX: provided technical support in echocardiographic diagnosis and serum sample collections; JuX: was involved in discussion on experimental design and contributed to the writing of the paper; ZS and YB: designed the experiments and contributed to the writing of the paper.

### Conflict of interest statement

The authors declare that the research was conducted in the absence of any commercial or financial relationships that could be construed as a potential conflict of interest.
